# Supplementary figures and images for: Population structure and antimicrobial resistance patterns of Salmonella Typhi isolates in urban Dhaka, Bangladesh from 2004 to 2016
Source: PLoS Negl Trop Dis. 2020 Feb 27;14(2):e0008036. doi: 10.1371/journal.pntd.0008036 (PMC7064254; doi:10.1371/journal.pntd.0008036)

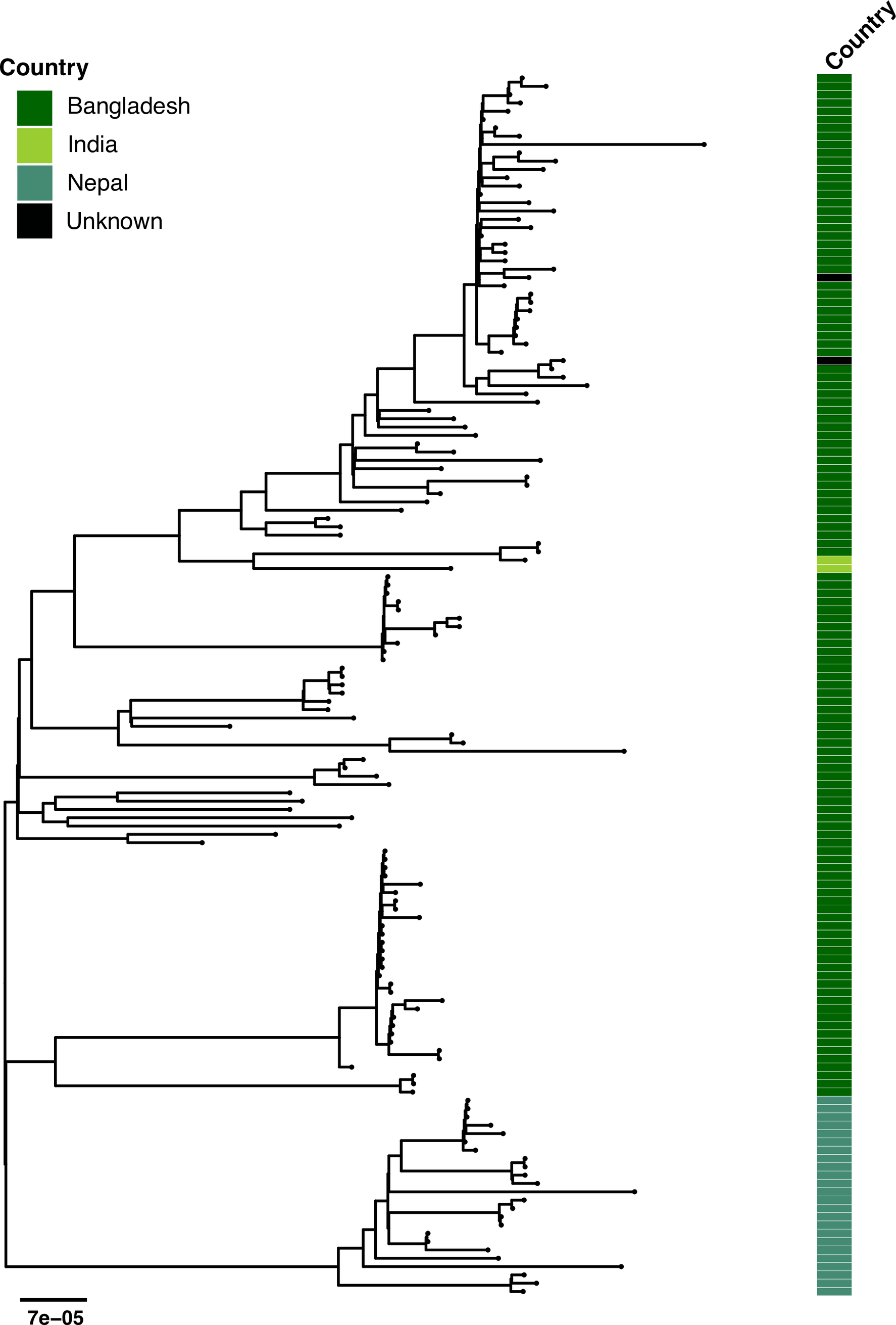

Supplement: S1 Fig — Maximum likelihood phylogeny of genotype 3.3.2 isolates. Colored bar indicates country of origin as per the inset legend. (TIF) [file pntd.0008036.s001.tif]

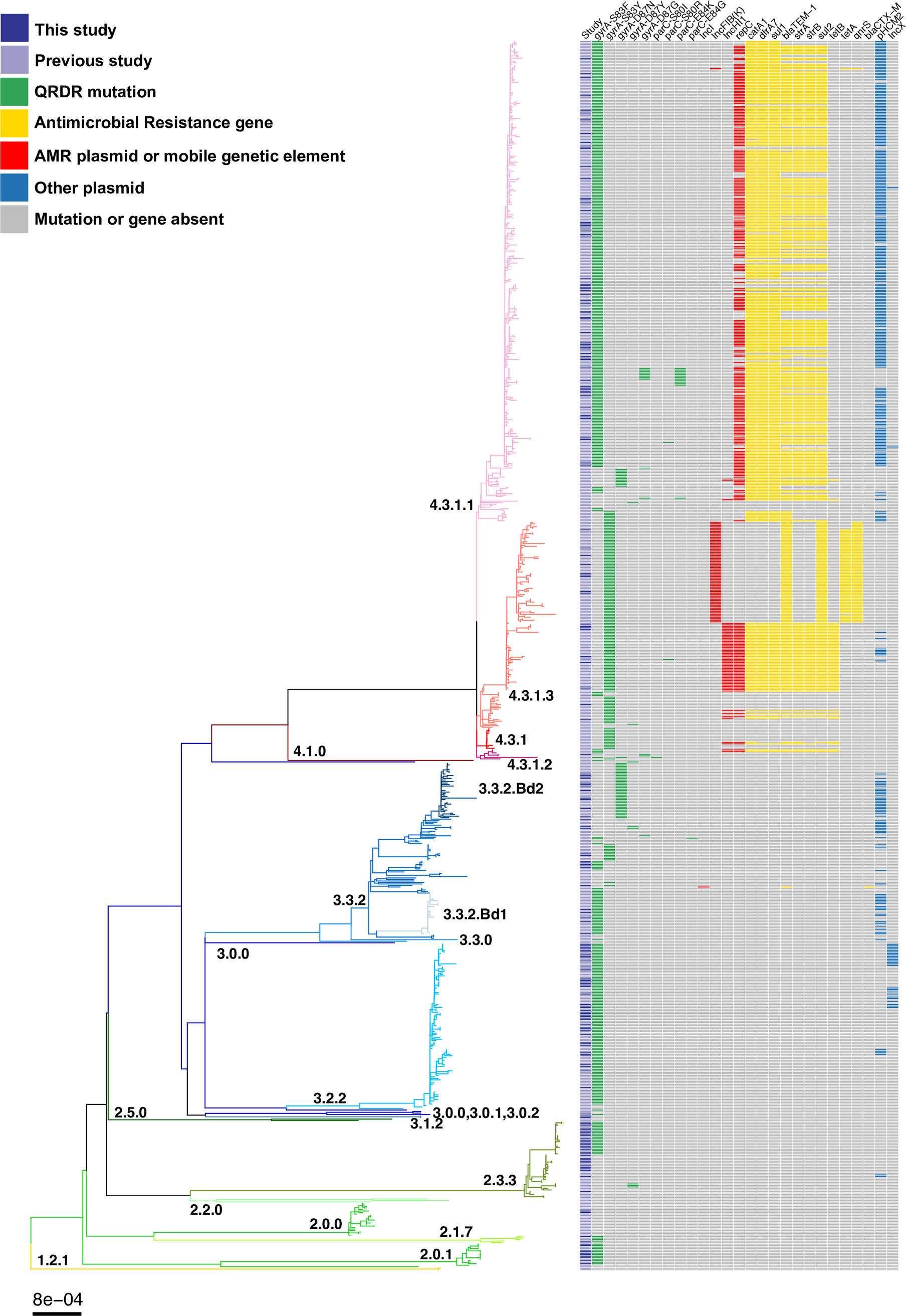

Supplement: S2 Fig — Branches are colored by genotype as labelled, the heatmap the molecular determinants of antimicrobial resistance and the presence of plasmids colored as per the inset legend. (TIF) [file pntd.0008036.s002.tif]
