# Supplementary material for: Population structure and antimicrobial resistance patterns of Salmonella Typhi isolates in urban Dhaka, Bangladesh from 2004 to 2016
Source: PLoS Negl Trop Dis. 2020 Feb 27;14(2):e0008036. doi: 10.1371/journal.pntd.0008036 (PMC7064254; doi:10.1371/journal.pntd.0008036)
Supplement: S4 Table — (DOCX) [file pntd.0008036.s006.docx]

**Table S4.** Genotypes of 818 *S.* Typhi isolates from Bangladesh.

| **Genotype** | **No. of isolate (%)** |
| --- | --- |
| 1.2.1 | 2 (0.25%) |
| 2.0.0 | 23 (2.81%) |
| 2.0.1 | 15 (1.83%) |
| 2.1.7 | 5 (0.61%) |
| 2.2.0 | 3 (0.37%) |
| 2.3.3 | 51 (6.24%) |
| 2.5.0 | 2 (0.25%) |
| 3.0.0 | 2 (0.25%) |
| 3.0.1 | 3 (0.37%) |
| 3.0.2 | 1 (0.12%) |
| 3.1.2 | 2 (0.25%) |
| 3.2.2 | 110 (13.5%) |
| 3.3.0 | 2 (0.24%) |
| 3.3.2 | 117 (14.3%) |
| 4.1.0 | 1 (0.12%) |
| 4.3.1 | 14 (1.71%) |
| 4.3.1.Bd | 138 (16.9%) |
| 4.3.1.1 | 320 (39.1%) |
| 4.3.1.2 | 7 (0.86%) |
